# Surveillance and Genetic Analysis of Jamestown Canyon Virus in New York State: 2001–2022

**DOI:** 10.4269/ajtmh.23-0392

**Published:** 2023-10-09

**Authors:** Kiet A. Ngo, Joseph G. Maffei, Cheri A. Koetzner, Steven D. Zink, Anne F. Payne, P. Bryon Backenson, Jennifer L. White, Alan P. Dupuis, Laura D. Kramer, Alexander T. Ciota

**Affiliations:** ^1^ New York State Department of Health, The Arbovirus Laboratory, Wadsworth Center, Slingerlands, New York;; ^2^ New York State Department of Health, Bureau of Communicable Disease Control, Albany, New York;; ^3^ Department of Biomedical Sciences, School of Public Health, State University of New York at Albany, Rensselaer, New York

## Abstract

Jamestown Canyon virus (JCV) (Peribunyavirdae; *Orthobunyavirus*) is a mosquito-borne pathogen endemic to North America. The genome is composed of three segmented negative-sense RNA fragments designated as small, medium, and large. Jamestown Canyon virus is an emerging threat to public health, and infection in humans can cause severe neurological diseases, including encephalitis and meningitis. We report JCV mosquito surveillance data from 2001 to 2022 in New York state. Jamestown Canyon virus was detected in 12 mosquito species, with the greatest prevalence in *Aedes canadensis* and *Anopheles punctipennis*. Detection fluctuated annually, with the highest levels recorded in 2020. Overall, JCV infection rates were significantly greater from 2012 to 2022 compared with 2001 to 2011. Full-genome sequencing and phylogenetic analysis were also performed with representative JCV isolates collected from 2003 to 2022. These data demonstrated the circulation of numerous genetic variants, broad geographic separation, and the first identification of lineage B JCV in New York state in 2022.

Jamestown Canyon virus (JCV) (Peribunyavirdae; *Ortho*bunyavirus) belongs to the California serogroup of viruses that are known to cause human disease, including La Crosse virus (LACV) and Snowshoe hare virus (SSHV).^1^ The genome is negative-sense, single-stranded RNA composed of three segments—small (S), medium (M), and large (L)—that are approximately 1, 4.5, and 7 kilobases in size, respectively. The S segment encodes the nucleocapsid, whereas the M segment encodes the two structural glycoproteins; the L segment encodes the RNA-dependent RNA polymerase.

Jamestown Canyon virus was originally isolated from *Culiseta inornata* collected in Jamestown, Colorado, in 1961 and has since been isolated throughout North America.^2^^,^^3^ The virus has been found in numerous mosquito species, and white-tailed deer (WTD) (*Odocoileus virginianus*) have been implicated as the primary amplifying host.^4^^,^^5^ Approximately 55% of hunter-harvested WTD throughout New York state between 2007 and 2015 were found to be seropositive for JCV.^6^ Jamestown Canyon virus can be transmitted by mosquitoes both vertically and horizontally.^7^

Although most JCV infections in humans are generally asymptomatic or result in a mild febrile illness, severe infections can lead to encephalitis or meningitis. Neuroinvasive cases have been reported throughout the United States and Canada.^8^^,^^9^ In the past 6 years, there have been at least seven deaths linked to JCV infection in humans.^8^

We tested mosquito pools collected in New York state for JCV from 2001 to 2022, and we completed full-genome sequencing and phylogenetic analysis for 32 JCV isolates collected from 2003 to 2022.

Adult mosquitoes were collected using CDC light traps, identified by species, pooled (10–60 individuals), and processed as described previously.^10^ RNA extraction was performed as described previously^10^ and was tested for JCV by TaqMan real-time reverse transcription–polymerase chain reaction (RT-PCR) with the primer pairs (5′-GTC TGG TCG AGT GTG ATA TAC G-3′) and (5′-CAG CAC AAA TCC GGT TAC AG-3′), and probe (5′-/56-TAMN/CCG GCA CTA CAG TTA AAT CTG GAT GGT/3IAbRQSP/-3′). These primers and probe do not detect lineage B; however, all mosquito pools besides *Culex pipiens* and *Culex restuans* (bird feeders that are unlikely to be infected with JCV) were inoculated onto mammalian cell cultures for virus isolation. All cultures displaying pathology consistent with viral infection were then harvested and identified using molecular testing and sequencing. This pipeline includes standard RT-PCR with generic Orthobunyavirus primers (5-ATGACTGAGTTGGAGTTTCATGATGTCGC-3′ and 5′-TGTTCCTGTTGCCAGGAAAAT-3′). All amplified products were subjected to sequencing at the Wadsworth Center Advanced Genomic Technologies Core (WCAGTC) followed by identification using the Basic Local Alignment Search Tool available through the National Center for Biotechnology Information.^11^

Infection rates, defined as the number of infected mosquitoes per 1,000, were calculated by the maximum likelihood estimation method using an excel plug-in program developed by Dr. Brad Biggerstaff.

Coding regions of the S, M, and L segments were amplified (primers available upon request) using one-step superscript III RT-PCR with platinum Taq (Life Technologies, Carlsbad, CA) according to the manufacturer’s instructions. The products were purified for next-generation sequencing at the WCAGTC. Briefly, library preparations were performed using the Nextera XT kit (Illumina, San Diego, CA). The sequencing was performed on the MiSeq Illumina platform, resulting in 250-bp paired-end reads. Full coding sequences for the S, M, and L segments were aligned, and phylogenetic trees were generated in Geneious (version 11.1.5; Geneious Prime, San Diego, CA) using PhyML with the Jukes-Cantor substitution model. The robustness of the nodes was evaluated by performing 500 bootstrap replicates. Trees were rooted to Inkoo virus S, M, and L segments (GenBank nos. KT288286, KT288285, and KT288284, respectively). Genetic distances were calculated using Mega10 X (Pennsylvania State University).

From 2001 to 2022, we tested 75,035 mosquito pools comprising approximately 2.45 million individuals, primarily representing five genera: *Aedes *(*Ae.*),* Coquilletidia *(*Cq.*),* Culiseta *(*Cs.*),* Culex *(*Cx.*), and *Anopheles *(*An.*). The infection rates were calculated by mosquito species (Figure 1A) and year (Figure 1B). Positive pools for the JCV were detected in 16 mosquito species: *Ae. cantator* (*n *= 1), *Ae. sollicitans* (*n* = 1), *Ae. japonicus* (*n *= 1), *Ae. communis* group (*n *= 2), *Ae. triseriatus* (*n *= 2), *Ae. sticticus* (*n *= 2), *Cs. melanura* (*n* = 4), *An. quadrimaculatus* (*n *= 6), *Ae. trivittatus* (*n *= 8), *Ae. vexans* (*n *= 9), *Ae. stimulans* group (*n *= 18, *Ae. stimulans*,* Ae. excrucians*,* and Ae. fitchii* combined), *An. punctipennis* (*n *= 25), *Cq. perturbans* (*n *= 30), and *Ae. canadensis* (*n *= 42). The highest infection rates were observed in *An. punctipennis* (0.57) and in the *Ae. stimulans* group (0.47), followed by *Ae. canadensis* (0.15). *Aedes canadensis* and *An. punctipennis* have been associated with many viruses, and their feeding preferences strongly affect their potential for human disease transmission.^12^ *Aedes canadensis* is a mammalian feeder that has been implicated as a bridge vector of viruses associated with human disease, including the Eastern equine encephalitis virus, LACV, and SSHV.^13^^,^^14^ In New York state, *An. punctipennis* and *An. quadramaculatis*, both mammalian feeders, have been implicated recently in driving increased transmission of Cache Valley virus,^15^ another medically important *Orthobunyavirus*.^16^

**Figure 1.**
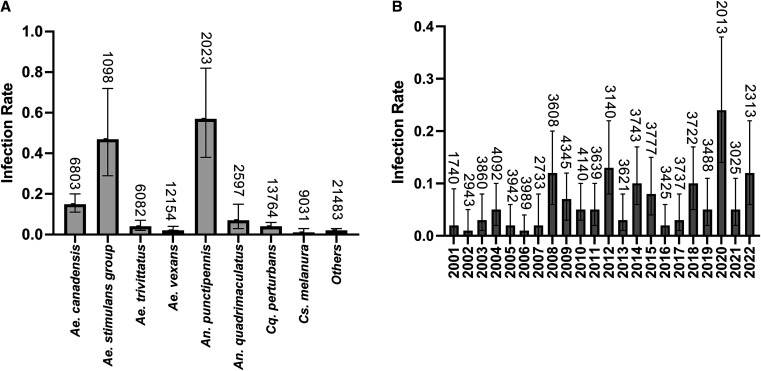
Jamestown Canyon virus infection rates from 2001 to 2022 in New York state. Infection rates were calculated by the maximum likelihood estimation method using a program developed by Dr. Brad Biggerstaff by (**A**) mosquito species and (**B**) year. The infection rate is defined as the number of infected mosquitoes per 1,000 tested. The numbers above the bars represent the total number of pools tested. Error bars represent 95% CIs intervals. Others: *Aedes *(*Ae.*)* communis* group *Ae. sticticus*,* Ae. japonicus*, *Ae. triseriatus*,* Ae. sollicitans*, and *Ae. cantator*.

The mean annual JCV infection rate from 2001 to 2022 was 0.06, with significant yearly variation (0.01–0.24) (Figure 1). Above-average infection rates (> 0.06) were observed in 2008, 2009, 2012, 2014, 2015, 2018, 2020, and 2022, with the highest in 2020 (0.24). The mean infection rate from 2012 to 2022 (0.09) was significantly greater than the mean infection rate from 2001 to 2011 (0.04; χ^2^ test with Yates’ correction, *P* = 0.0003). At least one JCV-positive mosquito pool has been detected since 2001, with the highest number of positive pools in 2020 (*n *= 16). Human cases of JCV have been reported to the CDC since 2012, yet mosquito infection rates in New York state are not well correlated with reported cases.^9^ For example, no cases were reported in New York state in 2020, when the highest infection rate was measured, yet three cases were reported in 2013, when a relatively low infection rate was measured. It is unclear how accurately reported cases reflect regional JCV burdens, given that most infections are undiagnosed. More comprehensive serosurveys could help clarify the relationship between JCV prevalence in mosquitoes and spillover to humans.

We selected 32 JCV isolates for sequencing (Table 1), representing different mosquito species and locations throughout our 22-year surveillance period. Our phylogenetic analyses also included 15 previously sequenced isolates from Connecticut (CT): CT1044, CT1627, CT2989, CT339, CT4078, CT4095, CT1262, CT2286, CT23, CT3573, CT3682, CT810, CT1064, CT4148, and CT4473.^17^ Phylogenies of the S, M, and L segments showed no evidence of strong temporal clustering, yet suggested broad geographic clustering (Figure 2A-D). Specifically, we identified the existence of a distinct cluster within lineage A in which all southern New York strains grouped together with Connecticut strains. Additional well-supported clusters comprising predominately western and central/northern strains were also identified, yet more mixing was apparent among these groups. The JCV L segment showed the strongest regional clustering (Figure 2D). All New York state JCV isolates were grouped into lineage A, except for one isolate (JCV 278) from 2022 that clusters with lineage B Connecticut isolates. This represents the first identification of lineage B in New York state, although it has been detected in previous years in Connecticut^18^ and Massachusetts.^19^ We observed disagreement among segment phylogenies with one central New York state JCV isolate: JCV9. The JCV9 M and L segments grouped with other central New York strains, whereas the JCV9 S segment grouped with the southern cluster. These data suggest the possibility of reassortment among clusters, which could drive further genetic and phenotypic diversification. Reassortants leading to new viral strains with consequences for human disease have been well documented for Orthobunyaviruses.^17^^,^^20^

**Table 1:** Jamestown Canyon virus strains used for genetic analysis

Year	Mosquito species	County (region)	Strain
2003	*Coquillettidia (Cq.) perturbans*	Westchester (southern)	JCV02
2004	*Aedes (Ae.) canadensis*	Oneida (central)	JCV04
2004	*Ae. stimulans* group	Clinton (northern)	JCV05
2005	*Ae. trivittatus*	Cattaraugus (western)	JCV06
2006	*Ae. canadensis*	Onondaga (central)	JCV07
2007	*Ae. triseriatus*	Westchester (southern)	JCV08
2007	*Ae. canadensis*	Onondaga (central)	JCV09
2008	*Anopheles (An.) punctipennis*	Putnam (southern)	JCV10
2008	*Ae. vexans*	Oneida (central)	JCV11
2009	*Ae. canadensis*	Chautauqua (western)	JCV12
2009	*Ae. stimulans* group	Erie (western)	JCV13
2010	*Ae. stimulans* group	Erie (western)	JCV14
2010	*Ae. canadensis*	Westchester (southern)	JCV15
2011	*Ae. triseriatus*	Westchester (southern)	JCV16
2011	*An. punctipennis*	Rockland (southern)	JCV17
2012	*Cq. Perturbans*	Onondaga (central)	JCV18
2012	*Ae. canadensis*	Oswego (central)	JCV19
2013	*Ae. canadensis*	Madison (central)	JCV21
2014	*An. punctipennis*	Rockland (southern)	JCV22
2014	*Ae. canadensis*	Onondaga (central)	JCV23
2015	*Ae. sticticus*	Cattaraugus (western)	JCV25
2016	*An. punctipennis*	Cattaraugus (western)	JCV26
2016	*Ae. canadensis*	Erie (western)	JCV27
2017	*An. quadrimaculatus*	Cattaraugus (western)	JCV28
2017	*Ae. vexans*	Suffolk (southern)	JCV29
2018	*An. punctipennis*	Chautauqua (western)	JCV30
2018	*An. punctipennis*	Erie (western)	JCV31
2019	*Cq. Perturbans*	Orange (southern)	JCV32
2019	*Ae. stimulans* group	Erie (western)	JCV33
2022	*Ae. canadensis*	Suffolk (southern)	JCV278
2022	*Ae. canadensis*	Madison (central)	JCV060
2022	*Cq. Perturbans*	Onondaga (central)	JCV333

**Figure 2.**
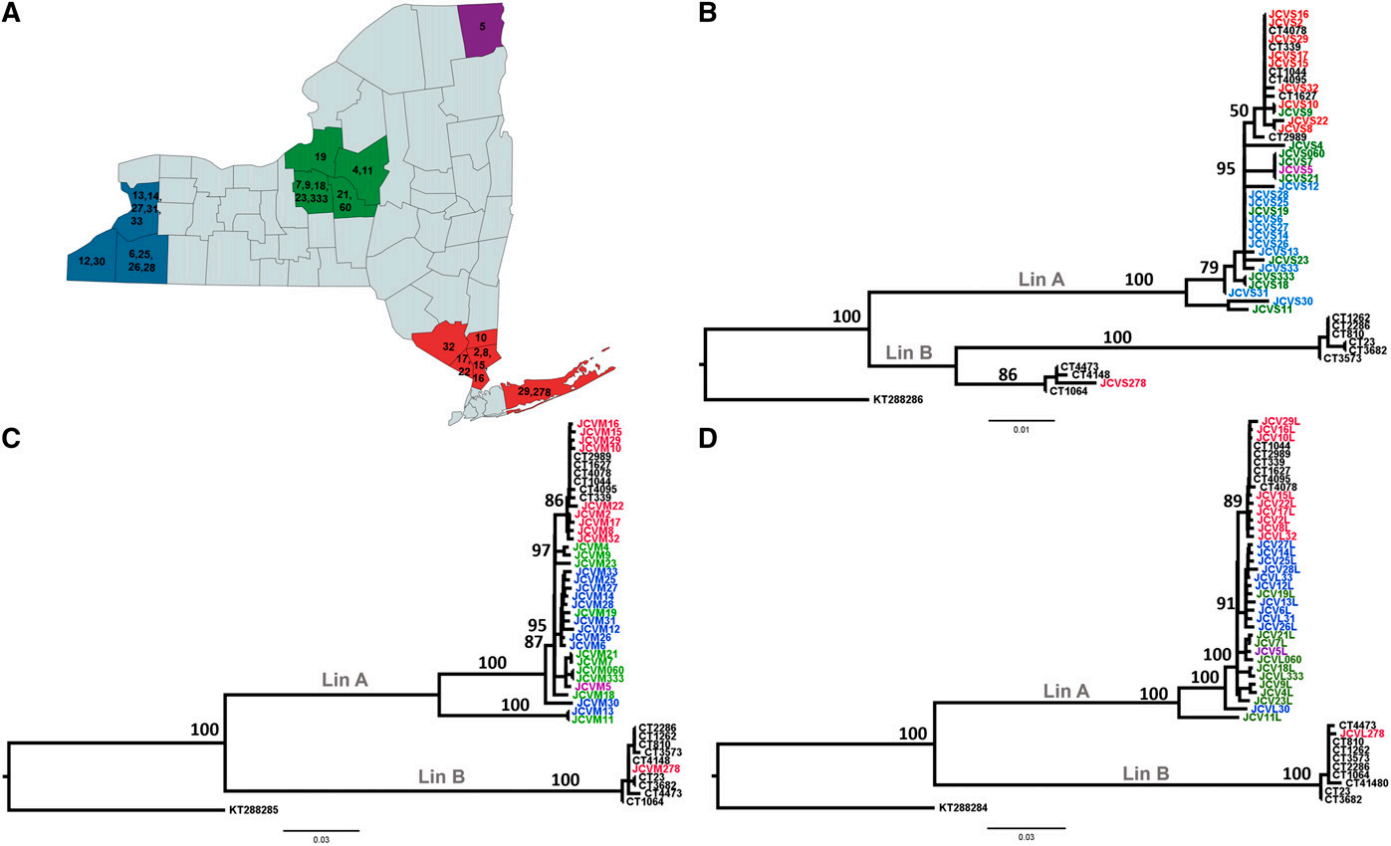
Phylogenetic analysis of the Jamestown Canyon virus in New York state. (**A**) Full-genome sequencing was completed for 32 isolates collected from four regions in New York state, including southern (red), northern (purple), central (green), and western (blue). Maximum likelihood analysis of the complete coding regions of (**B**) small, (**C**) medium, and (**D**) large segments was completed together with previously sequenced isolates from Connecticut (CT). The robustness of the nodes was evaluated by performing 500 bootstrap replicates. The trees were rooted to Inkoo virus small, medium, and large segments (GenBank nos. KT288286, KT288285, and KT288284, respectively). The colors of individual taxa correspond to regions of isolation. Well-supported bootstrap values of major nodes are shown on trees together with previously identified designations of lineage A (Lin A) and lineage B (Lin B).

The mean genetic distance, defined as the number of nucleotide substitutions per site, was calculated for each segment. The genetic distances of the S, M, and L segments are 0.006, 0.039, 0.010 within lineage A, and 0.030, 0.004, and 0.003 within lineage B, respectively. The mean genetic distances between the two lineages for the S, M, and L segments are 0.073, 0.141, and 0.135, respectively. Within lineage A (0.039), and between lineage A and B (0.141), there were more base substitutions per site in the M segment than in the S and L segments. Interestingly, in lineage A, the S segment was the most conserved (0.006), but was the most divergent in lineage B (0.030). Further studies are needed to understand more fully the consequences of within- and between-lineage genetic variability for virus transmission and disease, which are currently not well defined for JCV.

## Financial Disclosure

This work was funded in part by the CDC Cooperative (Agreement no. U01CK000509).
